# Barriers and facilitators to implementing advanced HIV disease screening at a secondary referral hospital -Malawi: a convergent parallel study

**DOI:** 10.1186/s12913-023-10009-5

**Published:** 2023-09-20

**Authors:** Brany Mithi, Agatha Bula, Lester Kapanda, Fatsani Ngwalangwa, Evanson Z. Sambala

**Affiliations:** 1 Department of Public Health, School of Global and Public Health, Kamuzu University of Health Sciences (KUHeS), P/Bag 360, Chichiri, Blantyre 3, Malawi; 2 University of North Carolina (UNC) Project, Lilongwe, Malawi; 3 Elizabeth Glaser Pediatric AIDS Foundation (EGPAF), Lilongwe, Malawi

**Keywords:** ART, People living with HIV, Advanced HIV Disease (AHD), Convergent parallel study

## Abstract

**Background:**

Malawi continues to register HIV/AIDS mortality despite increased expansion of ART services and as well as advanced HIV screening as outlined in the 2020 -2025 Malawi National HIV Strategic Plan (NSP). This study aimed to explore factors influencing the implementation of the advanced HIV disease (AHD) screening package at Rumphi District Hospital, Malawi.

**Methods:**

We conducted a mixed method, convergent study at a secondary referral hospital with 8 659 clients on ART. Guided by a consolidated framework for implementation research (CFIR) we conducted semi-structured Interviews with healthcare professionals, purposively selected from various key departments that were actively involved in AHD screening. Transcripts were organized and coded using NVivo 12 software with thematically predefined CFIR constructs. Newly HIV-positive client records extracted from ART cards (July –Dec, 2021) were analyzed using STATA 14 software.

**Results:**

One hundred one ART records met inclusion criteria for review and analysis of which 60% (*n* = 61) of the newly diagnosed HIV clients had no documented results for CD4 Cell count. Barriers to AHD screening emerged from four major CFIR constructs: intervention complexity, communication, availability of resources and access to knowledge and information. The specific barriers included poor work coordination among implementers, limited resources to support the expansion of AHD screening, and knowledge gap among providers. External support from Ministry of Health implementing partners and the availability of committed focal leaders coordinating HIV programs emerged as major enablers of AHD screening package.

**Conclusion:**

The study has identified major contextual barriers to AHD screening including knowledge gap, poor communication systems and inadequate supporting resources. Improving uptake of AHD screening services would therefore require overcoming the existing barriers by adopting a comprehensive approach in developing barrier-tailored strategies.

## Background

Despite the significant progress in expanding access to antiretroviral therapy (ART) in Malawi, AIDS-related deaths plateaued over the recent years [1, 2]. HIV Epidemiological Estimates for Malawi indicate that 10,800 deaths were registered among 987,000 adults (15 + years) living with HIV in 2020 [3]. The prevailing HIV mortality rates have been linked to opportunistic infections such as Tuberculosis (TB), and Cryptococcol meningitis often associated with Advanced stage of HIV Disease [4]. The impact has been immense on the performance of healthcare systems in poorly resourced settings particularly in sub-Saharan Africa [5, 6].

The 2020/2021 Malawi Population based HIV Indicator Survey (MPHIA) findings reveal significant gains in the UNAIDS 95–95-95 targets in terms of ART treatment coverage (97.3%) and viral load suppression (96.9%) but lagging behind on the first target (88.3%) for HIV positive individuals who are aware of their status [1]. Most the HIV positive individuals unaware of their status present with Advanced HIV Disease (AHD) due to late HIV diagnosis [7, 8].

Following the roll-out of the AHD management package of care in Malawi, several people living with HIV have been diagnosed with opportunistic infections and linked to care, owing to the use of point of care rapid devices such as Urine LAM, Cryptococcol antigen (CrAg) and PIMA machine for CD4 cell count. Besides, high AIDS mortality rate due to OIs de-masked by immune-reconstitution inflammatory syndrome (IRIS) often common in the first month of ART initiation has gone down [8, 9].

The Malawi National HIV Strategic Plan (NSP) emphasize the need for scaling up screening for AHD in all antiretroviral therapy sites in order to reduce AIDS mortality [10]. This strategy compliments the 2017 WHO-recommended AHD package of care integrated in the Malawi HIV Clinical Management guideline [11]. WHO defines advanced HIV disease as CD4 Cell count of less than 200 cells/µL or HIV clinical stages 3 and 4 which are associated with life threatening conditions [12]. Considering the high prevalence of TB and Cryptococcal meningitis among clients with AHD, scaling up AHD screening provides an entry point to care for newly diagnosed HIV positive clients who are often asymptomatic [4].

However, the implementation of AHD screening in various healthcare facilities has not been smooth. The HIV coverage quality and impact network (CQUIN) project in Malawi singled out COVID-19 as the major obstacle to delivery of AHD package of care as it led to decreased in the number of PLHIV attendance and delays in AHD trainings for healthcare providers [2]. Studies conducted in tertiary referral hospitals have also established barriers at the system and healthcare providers’ level [9]. For instance, limited trained personnel and non-compliance to AHD guidelines have been reported as key challenges in the implementation of AHD screening leading to testing inconsistencies and poor identification of persons with AHD [8, 10, 13].

Although the Ministry of Health (MoH)’s response has largely focused on conducting mentorship and onsite supervisions as well as expanding AHD screening sites, without understanding the nature and root cause of the existing contextual barriers, interventions are likely to be ineffective. Currently, local data about contextual barriers from secondary referral facilities is missing. Most of the previous AHD studies focused on hospitalized patients in tertiary referral hospitals. Our study, therefore, sought to investigate factors influencing implementation of AHD screening package among newly HIV-diagnosed clients in one of the secondary referral facilities in Malawi. The findings will provide a framework for designing strategies for scaling up AHD screening and improving linkage to care of those newly diagnosed with HIV.

## Methods

### Study design

An exploratory convergent study design was used to explore the contextual factors and gain a more comprehensive understanding of the impact of the barriers to delivery of AHD screening services. Data was collected in two parallel phases involving qualitative method where in-depth interviews were conducted and the quantitative method where secondary data from ART and AHD laboratory register was reviewed. Data analysis qualitative and quantitative methods was done separately.

### Study setting

The study was conducted at Rumphi District Hospital, Northern Malawi. The district is predominantly rural, covering an area of 4,769 km.^2^, with a population of 128,360 people. At the time of data collection, the hospital ART Clinic had 8,659 clients on ART, with HIV adult prevalence in district going at 5.3% according to MPHIA survey findings of 2021 [1]. The facility was chosen because it had the highest number of newly HIV diagnosed clients most of which were reportedly not being screened for AHD using the CD4 Cell Count test [2]. AHD screening was collaboratively done by laboratory staff, clinicians and nurses, with all the three AHD point of care tests (CD4 Cell count, serum CrAg and Urine LAM) conducted in the main laboratory.

### Study population and sample size

**Fig. 1:**
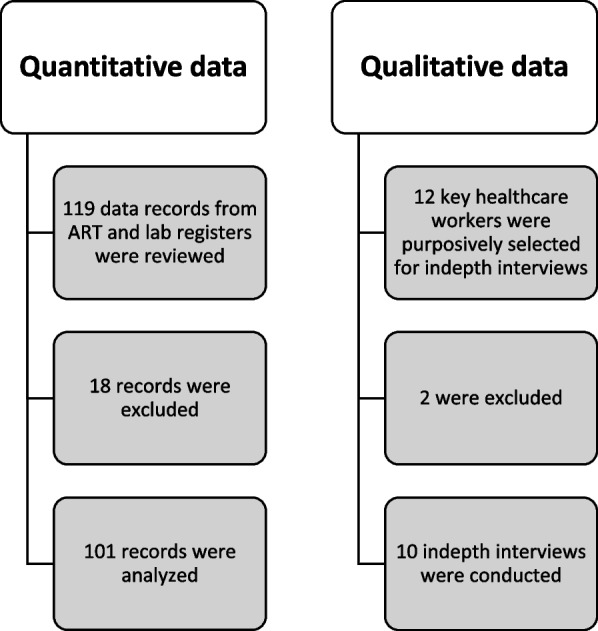
Data collection flow charts

Twelve homogenous, healthcare providers for AHD package of care were purposively selected from the hospital key departments with a goal of reaching data saturation with a small affordable sample size [14]. Facility in-charges assisted in the identification and recruitment of active participants in the implementation of the AHD screening. Only those who had worked in AHD screening sites for atleast 6 months were included. Lay cadres, students, interns and other temporary workers were not included. 119 ART records of newly diagnosed HIV positive clients, covering two quarterly months (July to December, 2021) was collected and reviewed. The Fig. 1 below summarize the data collection steps for the two highlighted methods.

### Framework of analysis

**Fig. 2:**
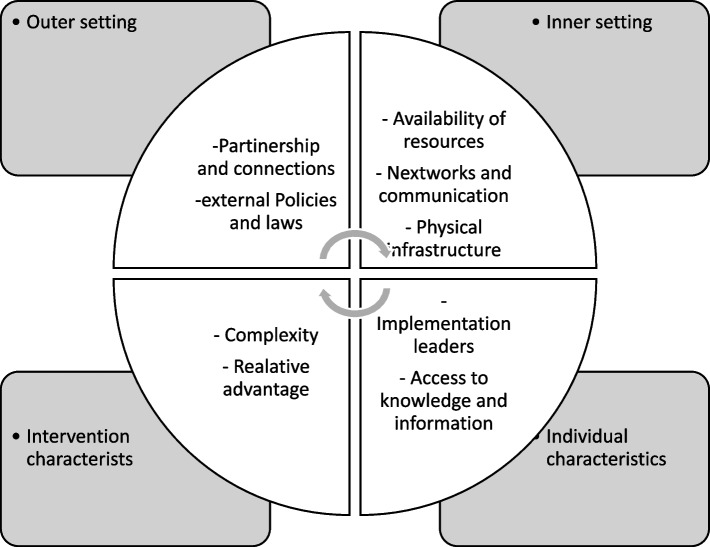
Major domains of the CFIR and their corresponding constructs

The Consolidated Framework for Implementation Research (CFIR) was used as an analytical framework to explore contextual barriers and facilitators from multiple levels within the healthcare setting [15]. The CFIR was selected because it provides a comprehensive understanding of the contextual determinants of health service innovations of proven effectiveness [10, 11]. The conceptual framework comprise five major domains; intervention characteristic, outer setting, inner setting, characteristic of individuals, and the process. CFIR has been used in various interventional studies and programmes [14–17]. The Fig. 2 shows CFIR domains and specific constructs which were used in this study [18].

### Data collection procedure

#### Data collection tool

Using the CFIR constructs (Fig. 2), an interview guide was designed and pre-tested during demonstration In-depth Interviews (IDIs) between research assistants and four other healthcare workers at the hospital. The aim was to determine the flow and appropriateness of the questions. Following the pre-test, some interview questions in the interview guide were modified. All providers who participated in the pilot testing were not part of the 12 selected healthcare providers for IDIs. Findings of the pre-test were treated as confidential research data.

#### In-depth interviews with healthcare workers

IDIs were conducted in English though participants were allowed to express themselves in Chichewa, the local language. Each IDI lasted for about 30–40 min. In addition to recording, field notes were written, highlighting important points and any new information emerging. Our initial target number for IDIs was 12 but we reached saturation at 10 where no new information emerged. All audio recordings were saved with participant Identity and securely stored in the password protected computer, waiting to be transcribed verbatim. IDIs in local language were audio recorded, transcribed and translated into English. Transcripts were sent to selected respondents to read and verify their responses. Both transcripts and their respective audios were later sent to an expert in qualitative research at UNC project –Malawi for further validation.

### Qualitative data analysis

Transcripts were analyzed using deductive method where data was coded according to the CFIR framework of analysis. During the initial phase of analysis, BM and AG independently reviewed the interview transcripts for accuracy. Following familiarization with data, a codebook was developed based on selected CFIR constructs to guide coding and categorization of data in the NVivo software version 12 (QSR International). Transcripts were entered into NVivo 12 where codes were indexed to sections of the transcripts. A thematic analysis was done in order to find patterns across the interview data. Finally, data was abstracted and interpreted, with direct quotations of respondents opinions included in the final report.

#### Secondary data collection (quantitative)

An experienced clerk working at ART clinic was recruited to help collect data for every newly HIV diagnosed client by reviewing ART records. A user friendly data collecting tool was designed to aid in collection of data records. The clerk cross-checked ART records with a laboratory AHD integrated register to verify if the CD4 Cell count tests were done as the first step in a spectrum of AHD screening tests. Data with missing valuables such as client name and age were excluded from the list. The final list was de-identified and assigned numbers in excel sheet.

### Quantitative data analysis

ART data records in the excel sheet were imported into STATA 14.0 software programme. Descriptive statistical analyses such as mean, median and interquartile range were generated to summarize demographic information of study participants. Monthly and quarterly proportions of individuals screened for AHD were also generated using STATA. Excel was used to create graphs for illustration of monthly proportions of AHD screened individuals. Findings are presented in form of tables and figures in the result section.

## Results

### Qualitative findings

#### Demographic characteristic of participants

Between April and May, 2022, 12 healthcare workers were recruited to the study and while 10 participated in semi-structured interviews. Table 1 summarizes the background characteristics of participants involved in the IDI. Among the 10 participants, four (40%) were clinicians, three (30%) laboratory scientists, and three (30%) nurses. Most of the participants were males (*n* = 8, 80%). Participants age range was 27 to 59 years (Mean 43.3, ± 8.2) with more than 10 years in civil service as healthcare workers (Mean = 15.5, ± 5.0). The majority possessed a Bachelor’s degree in health related discipline (50%, *n* = 5).

**Table 1:** Demographic characteristic of participants

**Sex**	**Age**		**Years of service**		**Profession**	**N0_**	**Level of Education**	**N0_**
**Male**								
08	Mean	43.3	Mean	15.9	Clinicians	4	Masters	0
**Female**								
02	SD	± 8.2	SD	± 5.0	Lab scientists	3	Degree	5
	Min	30	Min	10	Nurses	3	Diploma	4
	Max	57	Max	25			Certificate	1

### Perceptions of healthcare workers regarding AHD screening

This section present findings on healthcare workers knowledge and perceptions towards factors influencing the implementation of AHD screening package. Results have been presented either as barriers or facilitators mapped with most applicable CFIR constructs shown in Fig. 2 in the methodology section. Both barriers and facilitators are summarized in Table 2.

**Table 2:** Summary of barriers and facilitators

CFIR Domain	**CFIR Constructs/themes**	**Barrier/ Facilitator**	**Description**
A. Innovation characteristic	Critical incidents	Barrier	Demand for AHD screening services was low were disrupted by shortage of staff and clients restrictions as a result of COVID-19
	Complexity	Barrier	Healthcare providers perceived provision of AHD screening and diagnostic services as cumbersome and complex
	Relative advantage	Facilitator	Healthcare workers noted intervention benefits such as improved diagnosis & proper management of OIs as a result of AHD screening
B. Inner setting	Networks & communications	Barrier	Lack of functional ground telephone system affected work coordination among implementers
	Readiness to implementation		
	Availability of resources	Barrier	Facility lacked financial support to facilitate trainings for HCWs. Testing devices (PIMA machines) & tools were not adequate enough to expand testing to ART Clinic
	Structural characteristic		
	Physical infrastructure	Barrier	Distance between ART and Lab led to clients Loss to follow up and poor linkage to care
	Access to knowledge and information	Barrier	Most healthcare workers lacked knowledge of AHD screening as they were yet to be trained, hence low demand for the service
C. Outer setting			
	Partnerships & connections	Facilitator	The institution was well networked with external organizations e.g. NGOs, MoH
	Policies & laws	Facilitators	Though posters were not available. MoH policy documents such as guidelines and SOPs were present
D. Individual characteristics	Implementation leaders	Facilitator	Skilled focal leaders (coordinator, or team leader), with the responsibility to lead the implementation AHD package were available

### Contextual barriers

#### Outer setting: critical incidents, complexity of the intervention

COVID -19 pandemic was evaluated as a strong barrier to AHD screening from the outer setting domain under critical incidents. Participants highlighted that the pandemic disrupted screening as less clients reported for HIV testing and less staff were available for duties due to imposed restrictions.

*“So in terms of personnel I would say that they are not enough because with these upcoming diseases like COVID-19, most of the staff are taken up to be working in COVID-19 testing”*[RDH/Lab tech 3]

AHD screening process was described as complex as it involved various departments and multiple sequential tests which led to an increased patients waiting time. Additionally, results recording was reported to be a challenge, owing to a substandard integrated register for the three AHD rapid tests.

*“We have been given a CD4 register from the MoH, but it is missing these other tests (TB LAM, Serum CrAg). So if you are documenting these tests results, you will be using a separate improvised register which is cumbersome.”* [RDH/Lab tech 1].

#### Inner setting: readiness to implementation (Availability of supporting resources)

Respondents acknowledged the absence of supporting resources such as posters and testing algorithm which they said contributed to poor AHD screening. This was classified as a potential barrier to AHD screening. One respondent from ART clinic said:

*“The government has delayed to provide the posters because we should have pasted some in the wards for our friends who have less knowledge to understand what’s going on”* [RDH/Clinician 1].

Most respondents argued that it was difficult for other departments to start conducting the AHD diagnostic tests outside laboratory without the necessary laboratory equipment such as centrifuge and pipettes for sample processing as well as biosafety measures. Responding to the question as what could be the way forward, one of the nursing managers said:

*“The laboratory should continue providing the point of care tests (POCT ) up until the facility has enough resources to extend it to ART clinic”* [RDH/Nurse 2].

As a way of preparing the ART clinic for point of care testing (POCT) and to ensure that services are not interrupted due to staff engagements, a clinician working with lighthouse organization suggested the need to train lay cadres in conducting POCT at ART.

*“In other sites they trained the HDAs (HIV Diagnostic Assistants) and ART Clerks so it becomes easy when all nurses and clinicians are not available”* [LH/Clinician 2]

#### Inner setting: poor communication and networks

Physical barriers in the inner setting affected work coordination among teams involved in AHD screening. It was highlighted that the distance between ART clinic and the laboratory contributed to poor tracking of new ART clients undergoing AHD screening tests. One the providers argued that the current arrangement defeated the intended purpose of providing quick services to the clients which was against the policy that require testing services to be conducted at the ART clinic.

*“If the patient has to go to the lab to access testing services rather than within the ART clinic then it’s no longer point of care.”* [LH Nurse 3].

Absence of such communication systems affected both formal and informal information sharing practices which negatively impacted on work coordination among different working teams. Without ground telephone system in place, implementers used personal gadgets for communication.

*“Sometimes there are stockouts of some reagents in the laboratory. It’s very hard to know until you send a client and comes back without a test”* [RDH Nurse 1].

#### Individual characteristics: knowledge gap; self-efficacy

Respondents reported knowledge gap as a barrier claiming that providers who never attended formal trainings lacked confidence to conduct AHD screening. One of the participants argued that trained AHD providers were not enough in most of the screening sites:

*“In the male ward department, I think am the only one trained which means if am not available then we have problems* [RHD/Clinician 4].

### External facilitators 

#### Outer setting: relative advantage; external policy; partnerships and connection

Despite the complexity of the intervention, most participants perceived AHD screening package to have a relative advantage over the ordinary practice of care. They highlighted benefits such as improved diagnosis & proper management of opportunistic infections.

*“This kind of screening I think it was just delayed but it’s very essential and it has helped us a lot in reversing some of the deaths which were imminent*.” [RDH clinician 3]

The facility was found to be well supported externally. Technical staff from Lighthouse organization facilitated integrated HIV programme review meetings at district level while MoH facilitated integrated HIV mentorships programmes and national bi-annual AHD review meetings.

“*This week a team from Department of HIV/AIDs has been going around in different health facilities carrying out mentorship programmes”* [RDH Clinician 3]

### Internal facilitators

#### Inner setting: implementation leads

Respondents mentioned that the availability of two HIV programme coordinators for HIV Testing Services and ART, helped to spearhead the implementation of the AHD screening package.

*“As a trainer and Coordinator I make sure samples are collected adequately and run according to the specified guidelines. The ART coordinator makes sure that that new clients undergo CD4 test as the baseline test”* [RDH Coordinator 1].

### Quantitative results

#### Demographic characteristics

The Table 3 illustrate the background characteristics of the newly HIV-diagnosed clients from ART data records, covering two quarterly months in 2021. Out of 119 records, 101 met the inclusion criteria, with a balanced distribution of sex (M = 50 [49.5%], F = 51 [50.5%]). The median age was 33 (M = 35years, ± 11.9).

**Table 3:** Demographic characteristics of newly diagnosed ART clients

**1.** **AGE (Newly diagnosed ART clients)**			
Obs	101	Smallest	9
Male	50 (49.50%)	Largest	70
Female	51 (50.50%)	Sum of wgt	101
Median	33	Median	33
Mean	35.14851	Variance	140.6077
Std. Dev	11.85781		

Table 4 provide proportional estimates for clients who were screened for AHD. Of the 101 eligible clients, 61 (60%) of the newly HIV-diagnosed clients had no documented results for CD4 cell count as a baseline test for determining advanced stage of HIV. Both the third and fourth quarterly months had fewer clients screened for AHD {3^rd^ Q, 38% [19/50]; 4^th^ Q, 41% [21/51]}.

**Table 4:** CD4 count proportional estimates

**Proportion estimates for CD4 Count**				
2021				
	CD4 Done	CD4 not done	Total	
3^rd^ Quarter	19 (38%)	31 (62%)	50	
4^th^ Quarter	21 (41%)	30 (59%)	51	
	40	61	101	
	**Proportion**	**Std. Err**	**[95%**	**Conf. Interval]**
1 (CD4 done)	.3960396	.0489073	.3041413	.4959164
2 (CD4 not done)	.6039604	.0489073	.5040836	.6958587

**Fig. 3:**
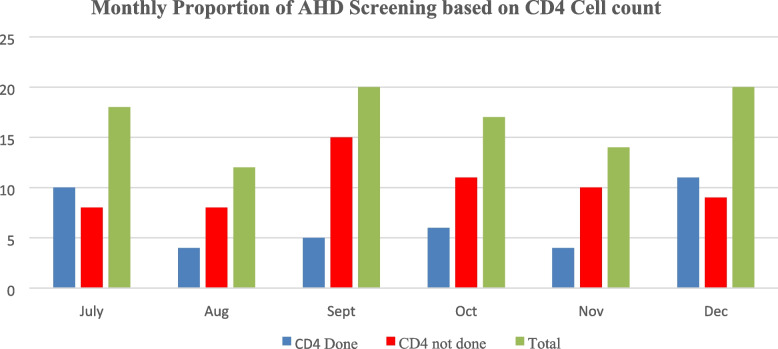
Clients screened for AHD using CD4 cell count

Figure 3 illustrate monthly proportions of newly diagnosed HIV positive client screened for AHD. Low AHD screening rate was registered for four consecutive months from August 33% (4/12), September 25% (5/20), October 35% (6/17) to November 30% (4/14). This could be attributed to contextual barriers to AHD screening as highlighted in Table 2.

## Discussion

This study sought to explore contextual factors that influence the implementation of AHD screening package at a secondary referral facility in Malawi using CFIR. 10 Key healthcare workers were interviewed whereas 101 ART data records were reviewed. Most of the barriers reported emerged from the inner setting and they related to complexity of the intervention due to multiple sequential steps involved, knowledge gap, poor communication systems and limited resources to support expansion of AHD screening among others.

Findings of the review of 101 ART data records for PLWH suggest that the implementation barriers had a significant negative effect on the delivery of AHD screening services provided at the laboratory. More than sixty percent of the eligible clients missed CD4 Cell count, a baseline test run before new clients are linked to treatment. This implies that most clients were not screened for TB and Cryptococcal meningitis using Urine LAM and serum CrAg respectively. Considering high number of cases of late HIV diagnosis presenting with AHD, newly HIV diagnosed individuals may have high risk of dying from life threatening conditions and possibly Immune reconstitution inflammatory syndrome (IRIS) after being initiated on ART. We describe the barriers that led to screening under coverage as well as the facilitators and proposed strategies for scaling up the WHO recommended AHD screening package of care.

Our findings suggest that lack of interpersonal-communication systems significantly contributed to poor work coordination among providers of AHD screening. All respondents from ART clinic highlighted similar communication challenges when dealing with laboratory staff. They further explained that laboratory staff were not communicating on time about stockouts and suspension of testing services, a practice that contributed to increased patient waiting time. It was established that that the facility had no any functional ground phone system nor WhatsApp group to facilitate information sharing and communication between the two crucial departments. Barriers of networks and communication within the implementing facilities often result in fragmented teams due to poor communication and frequent changes in personnel [19].

Interpersonal communication is an important facilitator of effective delivery of HIV interventions and therefore measured must be put in place to sustain it. Tailored cost-effective measures such as regular inter-departmental meetings and WhatsApp group forums provides a simple, readily available means of facilitating consultations between healthcare providers in the management of patients where formal communication systems are inefficient [19, 20].

Barriers due to incompatible work flow, congestion and as well as long distance between ART and laboratory departments were also reported. These contributed to long AHD screening pathway that led to clients lost to follow-up and poor linkage to care. As a result, clinicians and nurses at ART department resorted to providing ARVs to their new clients before AHD screening, a practice that was not conforming to national standard guidelines. Corresponding findings are reported in a study in the Eastern Africa where overcrowding, long waiting times and lack of resources emerged as prominent barriers to implementation [21]. To address the problem of long waiting time and loss to follow up, most respondents were of the opinion that AHD diagnostic services should be extended to ART clinic where the majority of the new ART clients report for care.

However, setting up a min-testing laboratory at the ART clinic like it is done in central hospital facilities, would require resources for biosafety and biosecurity infrastructure as well as devices for sample processing. In that regard, prior planning and budgeting should be considered. A recent study at a tertiary healthcare facility in Malawi estimated a capital investment cost of establishing and equipping an advanced HIV disease room for diagnostic tests to be U$10,708 [9]. This is affordable for MoH with the support from the U.S. President’s Emergency Plan for AIDs Relief (PEPFAR) which has recently focused on decreasing mortality among PLWH by addressing advanced HIV Disease and its associated opportunistic infections [22].

This study also established that healthcare providers with less knowledge of the intervention lacked confidence and self-efficacy when conducting AHD screening. This led to low screening coverage. As a result, many eligible clients were missed out as evidenced by the findings of ART record review where 60% had no CD4 Cell count results. Moreover, all screening sites had no AHD posters for reference. Such findings are in agreement with other programme evaluation studies that highlight inadequately trained providers as a stumbling block to service delivery [23, 24]. To address the knowledge gap and improve self-efficacy, formal AHD trainings are highly recommended.

Improving knowledge and information about an intervention through informal training is of great essence as well as it leads to improvement in provider’s compliance to guidelines [25]. As such, implementing health facilities are encouraged to make use of other learning platforms such as morning-handovers reporting and ground-ward rounds to impart knowledge among key implementers of AHD screening including students and interns. This would lead to improved provider’s self-confidence, uniformity and consistent quality of work through adherence to policy guidelines supporting the intervention [26].

In addition to formal and informal trainings, provision of posters displaying eligibility criteria as well as testing arigorithm may provide a reference for healthcare providers with less knowledge of the intervention. One advantage of using posters is that they are readily available, hence provides instant knowledge and awareness of the intervention to healthcare providers [27].

It is important to highlight that provider’s awareness of the interventional relative advantage emerged to be another facilitator of AHD screening. Respondents explained that trained healthcare providers perceived the AHD screening package of care to have more clinical benefits compared to the ordinary practice of care before the programme was rolled out in 2020. They further alluded that more deaths have been reversed with the coming of AHD diagnostic tests and treatment package. Currently, there is overwhelming evidence that AHD screening improves early diagnosis and management of opportunistic conditions, thereby contributing to general improvement of quality of life for PLWH who would otherwise succumb to death [2, 22, 28–30]. As more people present with AHD due to late HIV testing, screening for AHD and its associated life threatening conditions become the only gateway to appropriate care [4].

Regarding networks and partnerships, technical support from MoH and its implementing partners emerged to be strong enablers of AHD screening as they helped to fill in the gap of knowledge through capacity building. Respondents claimed that quarterly visits by MoH delegates conducting ART integrated supervisions improved overall facility performance in terms of screening and management of clients diagnosed with AHD and its associated life threatening conditions. HIV integrated review meetings as well as MoH led mentorship and supervision programmes are the drivers of implementation as they provide platforms for learning where implementers from different health facilities share successes and challenges faced in the implementation of AHD screening services.

Internally, the presence of focal persons facilitate efficient delivery of health interventions. In this study, programmes coordinators for HIV Testing Services (HTS), and ART showcased extensive knowledge of the AHD management package of care with high level of commitment. Focal leads spearhead the implementation process thereby simplifying complex interventional packages which involve multiple steps and departments at a facility level [31]. Globally, there is high demand for well-trained focal leaders in healthcare and advocacy, especially in countries with high HIV prevalence and limited human resource capacity [32, 33].

It is important to note that some of the implementation challenges faced can be partially addressed by integrating AHD screening services into the existing HIV testing services (HTS) and ART programmes which are sufficiently supported by externally and have already established structures for communication and expansion. Integration of related health programs reduces duplication of services, is cost-effective, and as well as efficient [34, 35].

### Study limitations

This study has potential limitations. Firstly, due to time and financial constraints, the study was not done on large scale involving multiple health facilities implementing the AHD screening. Different health facilities might present with different implementation challenges. Secondly, we did not include newly diagnosed-HIV positive clients for interviews to understand their experiences and challenges faced during AHD screening. Therefore, the findings of this study may not be generalizable considering the sample size. Nonetheless, the validity of the findings was partly achieved by the use of mixed methods design (qualitative and quantitative).

## Conclusion 

In this study, poor coordination among providers, knowledge gap and lack of supporting resources were identified as major barriers to AHD screening, affecting the quality of care given to clients being linked to ART for the first time. For low income countries such as Malawi, improving access and coverage of AHD screening would therefore require overcoming the existing barriers in the facility set-up. One of the ways is to adopt cost effective communication approaches for improving work coordination and to maintain good institutional partnerships with MoH implementing partners to ensure continued technical support towards AHD management package of care. The study findings provide an insight of common contextual barriers and further suggest comprehensive approaches for HIV programme implementers to adopt and design barrier-tailored strategies for optimizing AHD screening and diagnostic services in healthcare facilities.

### Recommendation

To maximize screening coverage, MoH must provide all the necessary resources to ensure that departments such as ART Clinic have sufficient supporting materials to independently conduct all tests for AHD screening. Besides, the use of CFIR framework of analysis in evaluation of such complex HIV interventional programs to assess implementation progress highly recommended. Future studies should focus on understanding clients social-economic barriers and as well as testing acceptability and feasibility of tailored strategies highlighted in this study.

## Data Availability

This datasets generated and or analysed during the current study are not publicly available to ensure the privacy and confidentiality of the participants but are available from corresponding author on reasonable request.

## Abbreviations

ART: Antiretroviral Therapy
AHD: Advanced HIV Disease
CFIR: Consolidated Framework for Implementation Research
CM: Cryptococcal Meningitis
COMREC: College of Medicine Research Ethics
CrAg: Cryptococcol Antigen
DHSS: Director of Health and Social Services
EGPAF: Elizabeth Glaser Pediatric Aids Foundation
HCWs: Health Care Workers
HIV: Human Immunodeficiency Virus
IRIS: Immune Reconstitution Inflammatory Syndrome
MHIRST: Malawi HIV Implementation Research Scientist Training program
MoH: Ministry of Health
MPHIA: Malawi Population based HIV Impact Assessment
PEPFAR: President’s Emergency Plan For AIDS Relief
PLHIV: People Living With HIV
SSA: Sub-Saharan Africa
TAT: Turn Around Time
TB: Tuberculosis
UNAIDS: United Nations Programme on HIV/AIDS
WHO: World Health Organization
